# Limitations of radiosensitization by direct telomerase inhibition to treat high-risk medulloblastoma

**DOI:** 10.3389/fonc.2023.1104670

**Published:** 2023-01-18

**Authors:** Satarupa Sengupta, Shiva Senthil Kumar, Kathryn Bondra, Matthew Sobo, Xiaokui Mo, Rachid Drissi

**Affiliations:** ^1^ Division of Pulmonary, Critical Care, and Sleep Medicine, Department of Internal Medicine, University of Cincinnati College of Medicine, Cincinnati, OH, United States; ^2^ Center for Childhood Cancer, Nationwide Children’s Hospital, Columbus, OH, United States; ^3^ Greehey Children’s Cancer Research Institute, University of Texas (UT) Health San Antonio, San Antonio, TX, United States; ^4^ Department of Technical and Scientific Support, Diapharma, Cincinnati, OH, United States; ^5^ Center for Biostatistics, Ohio State University, Columbus, OH, United States; ^6^ Department of Pediatrics, The Ohio State University College of Medicine, Columbus, OH, United States

**Keywords:** telomerase inhibition, ionizing radiation, radiosensitization, high-risk medulloblastoma, therapy

## Abstract

Medulloblastoma (MB) is the most common malignant pediatric brain tumor. Previous studies have elucidated the genomic landscape of MB leading to the recognition of four core molecular subgroups (WNT, SHH, group 3 and group 4) with distinct clinical outcomes. Group 3 has the worst prognosis of all MB. Radiotherapy (RT) remains a major component in the treatment of poor prognosis MB but is rarely curative alone and is associated with acute and long-term toxicities. A hallmark of cancer cells is their unlimited proliferative potential which correlates closely with telomere length. The vast majority of malignant tumors activate telomerase to maintain telomere length, whereas this activity is barely detectable in most normal human somatic tissues, making telomerase inhibition a rational therapeutic target in the setting of cancer recurrence and therapy resistance. We and others have previously shown that short telomeres confer sensitivity to ionizing radiation (IR) suggesting that telomerase inhibition mediated telomere shortening will improve the efficacy of RT while minimizing its side effects. Here, we investigated the efficacy of the combination of IR with IMT, a potent telomerase inhibitor, in an *in vivo* model of group 3 MB. Our results indicate that although IMT inhibited MB telomerase activity resulting in telomere shortening and delayed tumor growth, the combination with IR did not prevent tumor recurrence and did not improve survival compared to the treatment with IR alone. Together, these findings suggest that the radiosensitization by direct telomerase inhibition is not an effective approach to treat high-risk pediatric brain tumors.

## Introduction

Telomeres are the physical ends of eukaryotic linear chromosomes and, in mammals, are composed of several kilobases of tandem TTAGGG repeats that are bound by the shelterin protein complex (1). With each cell cycle, telomeres shorten until they reach a critical length that triggers cellular senescence or apoptosis. This is counteracted by the activity of the telomerase enzyme. Human telomerase consists of at least two essential components, a protein catalytic subunit (hTERT) and an RNA template (hTERC) that contribute to the synthesis of telomeric repeats, thereby renovating telomeres. Telomerase activation, a feature of the vast majority of cancers, is essential for maintaining an immortal phenotype by conferring unlimited replicative potential, whereas in most normal somatic cells, this activity is not detectable, supporting the rationale of targeting telomerase and telomeres to treat cancer (2–4). We and others have shown that telomere shortening enhances sensitivity to ionizing radiation (IR) by altering the kinetics of the DNA damage response (5, 6). We previously conducted a molecular biology and phase II study of Imetelstat (IMT), a potent inhibitor of telomerase (7, 8) to estimate inhibition of tumor telomerase activity and efficacy in children with recurrent central nervous system (CNS) malignancies (9). The regimen proved intolerable and ineffective due, at least in part, to toxicities that prevented more frequent dosing of IMT to allow sustained inhibition of telomerase and tumor burden reduction.

Medulloblastoma (MB) is the most common pediatric brain tumor in the posterior cranial fossa, accounting for approximately 25% of all brain tumors in children (10). Previous studies have elucidated the genomic landscape of MB leading to the recognition of four core molecular subgroups (WNT, SHH, group 3 and group 4) with distinct clinical outcomes (10, 11). Group 3 MB overall displays the worst prognosis. Radiotherapy is a standard treatment in older children with group 3 MB but is rarely curative alone and is associated with acute and long-term toxicities (12–14). We have previously shown that over 90% of MB patients express *hTERT* and demonstrated telomerase activity (15). High expression levels were associated with worse progression free survival and overall survival. Group 3 patients had the highest *hTERT* expression. To test the efficacy of radiation therapy while minimizing its side effects, we investigated the efficacy of the combination of IMT, as a radiosensitizer, in *in vitro* and *in vivo* models of group 3 MB telomerase-positive stem-like cells derived from high-risk group 3 pediatric MB (10) (harboring *MYC* amplification). Our results indicate that although IMT inhibited tumor telomerase activity resulting in telomere shortening and delayed tumor progression, the combination with IR did not prevent tumor recurrence and did not improve survival compared to the treatment with IR alone. These findings indicate that the direct telomerase inhibition combined with IR has limited efficacy and new approaches utilizing this quasi-universal cancer target are required to treat high-risk pediatric brain tumors. This is the first report evaluating the combination of telomerase inhibition combined with IR in high-risk group 3 medulloblastoma.

## Materials and methods

### Primary tumor cell culture

High-risk group-3 medulloblastoma primary patient-derived neurospheres MB004 (*TP53* mutated, *c-MYC* amplified) (16, 17) were cultured in neurosphere stem cell media as previously described (18). Briefly, cells were cultured in tumor stem media in serum-free condition consisting of DMEM/F12, Neurobasal-A, B27 (Gibco); human-basic EGF, FGF (Shenandoah biotech); and Leukemia Inhibitory Factor (Millipore). For sphere formation assay, MB004 neurospheres were dissociated by TrypLE express (Gibco), and single cells were seeded in 96-well plate in serial dilution up to single cell per well. Sphere re-growth or self-renewal was monitored by microscopy. Cells were cultured in the presence of 10% FBS to test adherence and differentiation ability.

### Drug treatment

Imetelstat (GRN163L; Geron Corp.) was dissolved in 1X PBS to prepare a 282 µM stock solution for *in vitro* use, and 1 mg/mL stock for *in vivo* studies. Mismatch (MM) control oligonucleotide was prepared the same way as Imetelstat. After reconstitution, drug was aliquoted and stored in -20°C. *In vitro*, short-term treatments were conducted for 72 hours, and long-term studies were conducted for up to 6 weeks then cells were reseeded with fresh media (with or without Imetelstat). *In vivo*, Imetelstat (15 mg/kg) was intraperitoneally (i.p.) administered.

### Telomerase activity assay

Telomerase activity was assayed using the TRAPeze Telomerase Detection Kit (Millipore). Cell extracts were prepared according to the manufacturer’s protocol. A total of 50 to 100 ng of total protein was used to assess the telomerase activity.

### Telomere restriction fragment analysis

Telomere lengths were determined by Southern blot using the TeloTAGGG Telomere Length Assay Kit (Roche Diagnostics). Genomic DNA was extracted from Imetelstat treated or untreated MB004 cells or xenograft tissue using the Gentra Puregene kit (Qiagen). 1 μg genomic DNA was digested, separated by gel electrophoresis, and transferred to a charged nylon membrane. Hybridization and detection were carried out following the manufacturer’s instructions. Mean telomere length was determined by comparing the mean size or the maximum intensity of the smear relative to the molecular weight marker provided in the kit, using TeloTool version 1.3 (19). Genomic DNA with known telomere length supplied in the kit was used as positive control.

### Patient-derived mouse xenograft and treatments

Six-seven weeks-old athymic Ncr-*nu/nu* female mice (J:NU (*Foxn1^nu^/Foxn1^nu^*, The Jackson Laboratory) were subcutaneously injected in single flank with 10,000 MB004 cells as previously described (18). Ten days postimplantation, Imetelstat dosing was initiated intraperitoneally at 10 mg/kg, and 15 mg/kg doses twice a week along with a vehicle control (PBS). Tumors were measured every other day by slide calipers taking two longest tumor-diameters (length and width) perpendicular to each other and volumes were calculated by using the formula: (π/6) x d^3^, where d = mean diameter. For irradiation (IR) experiments, IR doses were started when the tumors reached the volume of 500 mm^3^ and were given in fractions of 2 Gy per day for five days (Monday – Friday) a week. Localized mouse irradiation device with shielding apparatus was used to deliver focal irradiation to the tumors as described elsewhere (20). All animal procedures were conducted according to our IACUC protocol (#IACUC2015-0066, CCHMC).

### Immunofluorescence and immunohistochemistry

Immunostaining was performed as described previously (18, 21). For immunofluorescence, primary antibodies used were against Ki67 (Abcam), GFAP (DAKO), γH2A.X (Cell Signaling), Nestin (Millipore), and/or TRF2 (NOVUS), at 1:500 (rabbit) or 1:200 (mouse) dilutions as applicable. Corresponding secondary antibodies (Alexa-Fluor 488- or 594-conjugated donkey anti-rabbit, or anti-mouse (1:500) (Jackson ImmunoResearch) were used for 1 hour and washed with TBS (x3) before mounting with DAPI (Vector Laboratories H1200). Images were captured on Nikon Eclipse Ti confocal microscope. Formalin-fixed paraffin-embedded (FFPE) tissue sections of MB004 patient-derived xenograft were used for histopathological staining (H&E and Synaptophysin IHC). Tissues were mounted with Permount (Fisher Scientific) and imaged by Nikon eclipse 80i microscope.

### Statistical analysis

Student’s *t-test* or multiple-way ANOVA were applied as required, and Kaplan Meier Survival Analysis was performed using the GraphPad Prism (version 7.02). Each *in vitro* experiment was repeated at least twice. Error bars represent standard deviation from different animals considered as biological replicates. Differences were considered significant at *P <0.05. Log-rank test was applied to compare the differences in event-free survival between treated or control groups *in vivo*. Response, recurrence, or treatment failure rates, and multiple comparison for the Log-rank test were performed by the Center for Biostatistics, The Ohio State University, and as described elsewhere (22, 23).

## Results

### Characterization of patient-derived high-risk group 3 medulloblastoma cells

**Figure 1:**
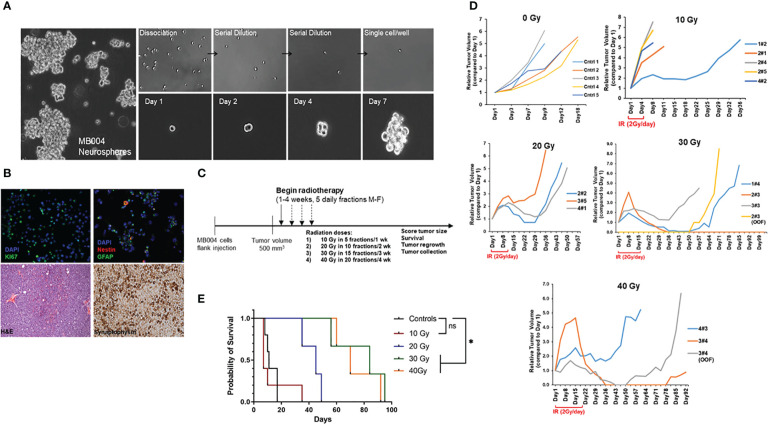
Characterization of patient-derived MB004 cells. **(A)**, images of neurospheres (left); serial dilution of dissociated single cells (right upper panel) and secondary sphere formation from a single cell cultured from day 1 to day 7 (right lower panel). **(B)**, upper panel, representative IF images of MB004 cells showing Ki67 in green (left), nestin and GFAP in red and green respectively (right). DAPI (blue) indicates nucleus staining. Lower panel, H&E (left) and synaptophysin staining (right) of MB004 patient-derived xenograft. **(C)**, scheme representing the experiment design to assess the effect of different IR doses in a flank xenograft model of MB004 cells. (M-F indicates Monday to Friday) **(D)**, tumor growth plots showing relative tumor volume (RTV) of MB004 PDX tumors untreated (0 Gy, n=5) or treated with 10 Gy (n=5), 20 Gy (n=3), 30 Gy (n=3), and 40 Gy (n=3) irradiation (IR). Duration of IR in each dosed group is indicated (2 Gy/day x 5 days per week). Each line indicates tumor growth per mouse. OOF indicates out of field of irradiation. **(E)**, survival plots of treated mice. * *p*< 0.0332; ns, not significant.

MB004 cells, derived from high-risk group 3 medulloblastoma patient were cultured in serum-free tumor stem cell media in a neurosphere culture system as described previously (18). This system allowed the selection of primary cancer stem-like cells, also known as the tumor initiating cells (TICs), representing a small sub-population of tumor, responsible for tumor growth and recurrence. We first tested the presence of cancer stem cell properties such as self-renewal, proliferation, neuronal origin, differentiation ability, and tumorigenicity. The patient-derived neurospheres, when dissociated into single cells, were able to form secondary neuropsheres demonstrating self-renewal property (**Figure 1A**). Further characterization of the cells detected the expression of markers of proliferation, neuronal precursor, and differentiation such as Ki67, nestin, and GFAP respectively (**Figure 1B**). Tumorigenic property of the primary cells was verified by the establishment of patient-derived xenograft (PDX), that retained high cellularity and evidence of neuronal origin evidenced by H&E, and synaptophysin staining respectively (**Figure 1B**).

### Treatment of group 3 medulloblastoma patient-derived xenograft with radiation reduced tumor growth, improved survival but did not prevent recurrence and subsequent animal death

To assess IR response, subcutaneous PDX tumors were irradiated with clinically relevant focal radiation given at 2 Gy/day, 5 days/week, Monday to Friday for 5, 10, 15 and 20 days to complete the cumulative doses of 10, 20, 30 Gy, and 40 Gy respectively (**Figure 1C**). Although initially responding to IR, most tumors recurred. Response to radiation and overall survival (up to 30 Gy) was directly proportional to the cumulative IR doses (**Figures 1D, E**). The 10 Gy group showed the least response with the majority of the mice showing no tumor regression. All mice in 20 Gy group showed either some stabilization or regression in tumor growth for 2 weeks after the last fraction of 2 Gy. However, all tumors regrew, and no complete regression was observed. In both 30 Gy, and 40 Gy groups, at least 1 out of 3 mice (33%) showed complete regression with no instance of recurrence. The remaining mice (~67%) in both groups showed tumor volume stabilization or sustained regression for 2 weeks after the last dose. Of note, tumor regrowth was either observed in the field of irradiation at primary injection site or in distant locations (OOF, out of field, **Figure 1D**). Taken together, group 3 MB004 patient-derived neurosphere cells grown in stem-cell media represented an appropriate medulloblastoma model retaining cancer stem-cell like properties and tumorigenicity *in vitro* and *in vivo* and radiation alone did not prevent tumor recurrence and animal death.

**Figure 2:**
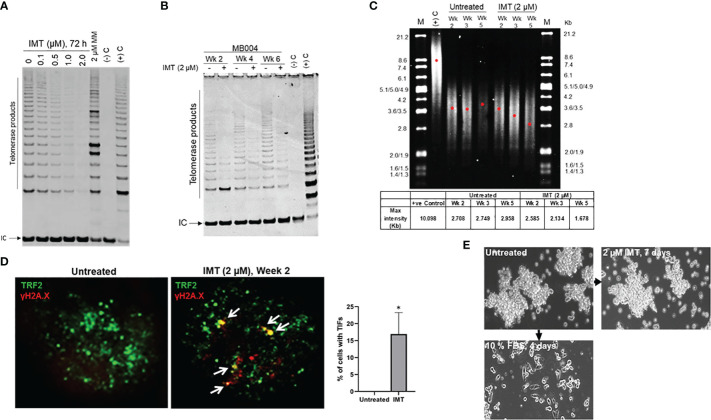
Prolonged telomerase inhibition leads to sustained telomere shortening. **(A)**, telomerase activity in MB004 cells, untreated and treated with Imetelstat (IMT) from 0.1 to 2 µM for 3 days, or with 2 μM mismatch (MM, IMT negative control) for 3 days. (-)C and (+)C, TRAP assay negative and positive controls respectively. IC, indicates internal PCR control. Telomerase products are indicated. **(B)**, TRAP assay evaluating telomerase activity in MB004 cells untreated or treated with 2 µM Imetelstat (IMT) for 2, 4, and 6 weeks. Wk, week, (-)C and (+)C, TRAP assay negative and positive controls respectively. IC, indicates internal PCR control. Telomerase products are indicated. **(C)**, telomere restriction fragment analysis of MB004 cells untreated or treated with 2 µM Imetelstat for 2, 4, and 5 weeks. along with positive (+ve) control. M, molecular weight marker with their associated sizes denoted in Kilobases (Kb). Each red dot denotes the maximum intensity of each smear in the respective lane indicative of the mean telomere length. Table (bottom) shows the maximum intensity values of the respective wells as indicated. wk, week.**(D)**, representative IF images of MB004 cells untreated and treated with 2 µM Imetelstat (IMT) for 2 weeks showing TRF2 (green, telomere marker), γH2AX (red, DNA damage marker). White Arrows indicate the colocalization of TRF2 and γH2AX, indicative of telomere dysfunction-induced foci (TIFs, yellow). The percentage of cells with TIFs were quantified. Error bars represent the SD from two different fields (10-15 cells/field). **(E)**, images of MB004 neurospheres untreated or treated with 2 µM Imetelstat (IMT) for 7 days or cultured in 10% FBS for 4 days as indicated. * *p*< 0.0332.

The hallmark of telomere dysfunction is the formation of DNA damage foci localized at telomeres called TIFs (telomere dysfunction-induced foci). TIFs are focal accumulations of DNA damage response factors such as ATMS1981-P, γH2AX, and 53BP1 at dysfunctional telomeres (24). We visualized TIFs by FISH combined staining using γH2AX colocalization with a telomere-specific PNA probe. As predicted, from MB group 3, MB004 cells displayed high levels of telomerase activity which was inhibited by IMT in a dose-dependent manner (0.1 to 2.0 μM) (**Figure 2A**). Long-term treatments (2, 4 and 6 weeks) of MB004 cells with IMT led to sustained telomerase inhibition, telomere shortening, and subsequent telomere damage evidenced by TIFs formation (**Figures 2B–D**). Furthermore, treatment with IMT did not affect the ability of MB004 cells to form neurospheres and did not induce their differentiation as shown in **Figure 2E** when cells were cultured in 10% serum, suggesting that IMT inhibited the canonical function of telomerase and did not affect the stemness of MB004 cells. Together, these results indicate that IMT treatment inhibits telomerase activity in MB004 stem-like cells leading to telomere shortening and telomere dysfunction-induced foci (TIFs) without affecting MB004 stemness, and prolonged treatments sustain this inhibition resulting in telomere shortening.

### Imetelstat delayed MB004 tumor progression, induced intratumoral telomerase inhibition and telomere shortening in patient-derived mouse xenograft model

**Figure 3:**
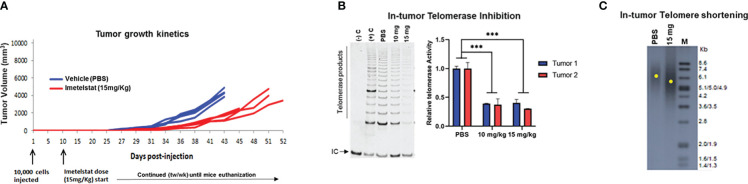
Evaluation of Imetelstat treatment in MB004 patient-derived xenograft (PDX). **(A)**, tumor growth kinetics of mice treated with PBS (vehicle) or 15mg/Kg Imetelstat. Each line denotes tumor growth per mouse. Mice were subcutaneously injected with 10,000 neurosphere cells. Imetelstat or PBS were intraperitoneally injected twice per week 10 days post-implantation for the indicated period of time. Each line denotes tumor growth per mouse. tw/wk, twice per week. **(B)**, left, representative TRAP gel image showing in-tumor telomerase activity levels in control (PBS, vehicle) and in 10 or 15 mg/Kg of Imetelstat treated MB004 PDX tumors collected at the end of the study. (-)C and (+) C are the negative and the positive controls of the TRAP assay respectively. IC, Internal PCR control; telomerase products are indicated. Right, corresponding plot showing quantitation of relative telomerase activity in two collected tumors. Error bars represent the standard deviation from two independent TRAP assays **(C)**, representative telomere restriction fragment analysis of MB004 PDX tumors treated with PBS (vehicle) or 15mg/Kg Imetelstat. M, molecular weight marker. Each yellow dot indicates the maximum intensity of each smear in the respective lane indicative of the mean telomere length. *** *p*< 0.0002.

Next, we tested the ability of IMT to inhibit telomerase activity *in vivo* in MB004 PDX and induce tumor growth inhibition. Athymic nude mice were subcutaneously injected with 10,000 MB004 cells, and IMT dosing was initiated intraperitoneally at 10 mg/kg and 15 mg/kg doses twice a week along with a vehicle control (PBS) group ten days postimplantation. Compared to the control group, IMT treatment delayed tumor growth (**Figure 3A**) and inhibited in tumor telomerase activity (**Figure 3B**) leading to a decrease in telomere length (**Figure 3C**).

### Imetelstat treatment combined with radiation delayed tumor recurrence but did not significantly improve survival outcome compared to radiation alone

**Figure 4:**
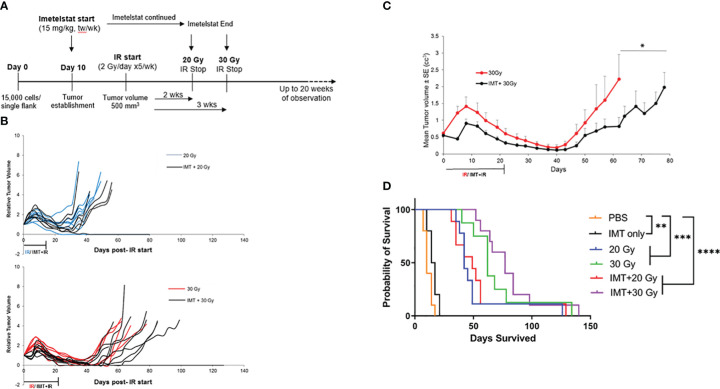
Limited effect of Imetelstat treatment combined with radiation in MB004 patient-derived xenografts. **(A)**, schematic diagram summarizing the workflow of combination treatment with Imetelstat (15mg/Kg) and IR (20Gy and 30Gy). tw/wk, twice per week. **(B)**, Relative tumor volumes (RTV) of MB004 PDX tumors treated with 20 Gy IR (blue) versus Imetelstat (IMT) + 20Gy IR (black) (upper plot); and 30Gy (red) versus IMT + 30Gy IR (black) (lower plot). Each line indicates tumor growth per mouse. **(C)**, Average tumor growth kinetics of 30 Gy IR only (red) and IR (30 Gy) + IMT (black) treated mice. The duration of IR or IMT + IR treatments are indicated. *P-*value is indicated, * *P*<0.05. Error bars represent the standard error mean between the tumor sizes from all the mice in their respective treatment groups, collected at each timepoint. **(D)**, corresponding survival plot of mice treated with vehicle (PBS), IMT, IR (20Gy or 30Gy), and IMT + IR (20Gy or 30Gy). * *p*< 0.0332; ** *p*< 0.0021; *** *p*< 0.0002, **** *p*< 0.0001.

The aim of our initial experiment with radiation (**Figure 1**) was to determine the IR doses to be used in combination with IMT. The minimal dose that induced minor response (25% tumor volume reduction in average) and partial response (50% volume reduction in average) were used with IMT. In the combination study, prior to IR, IMT was intraperitoneally administered, 15 mg/kg twice per week for two weeks. Tumors were then irradiated with the doses that induced minor and partial response, 20 and 30 Gy respectively (**Figure 1**). The objective of the combination treatment was to test the ability of IMT to sensitize tumors to IR by enhancing the minor and the partial response to IR and improving survival in comparison with IR alone. Thus, lowering the IR doses to achieve a complete or better response. We distributed athymic nude female mice into 6 groups (n=10 each): (a) untreated (vehicle), (b) IMT (15mg/kg), (c) 20 Gy IR, (d) 30 Gy IR, (e) IMT (15mg/kg) + 20 Gy IR, and (f) IMT (15mg/kg) + 30 Gy IR. Following injection of the MB004 cells subcutaneously, (IMT) treatment (15mg/kg, twice a week) was started intraperitoneally at day 10 upon tumor initiation evidence. When the tumors reached 500 mm^3^, the indicated radiation cumulative doses (2 Gy/day, 5 days a week) were started focally. IMT administration was continued until the last IR dose in both the IMT and combination groups. The mice were observed for tumor growth, regression, recurrence, and survival for up to ~20 weeks after the end of treatments (**Figure 4A**). Overall, there was no striking difference observed between the IR only and combination groups, as the majority (90%) of the tumors in all treatment groups recurred after an initial regression (**Figure 4B**). The regression was slightly prolonged in IMT+20 Gy and IMT+30 Gy groups compared to the corresponding cumulative doses alone. This regression was more pronounced in IMT+30 Gy group (**Figure 4B**). In IMT+30 Gy group 30% of the tumors (3 out of 10) showed a delayed recurrence compared to the 30 Gy tumors (**Figure 4B**). The IMT treated tumors were evaluated to confirm the in-tumor telomerase inhibition (**Figure S1**). Treatment with IMT and 30 Gy accentuated telomerase inhibition compared to IMT alone. Interestingly, IR treatment inhibited telomerase activity as previously shown (25, 26). When the mean tumor volumes were compared, IMT+30 Gy group showed a better regression curve and a significant delay in the recurrence (**Figure 4C**). However, both groups eventually reached exclusion criteria (due to tumor burden). Importantly, the survival plot did not reveal any significant improvement of survival benefit in the IMT+IR groups compared to the IR only (**Figure 4D**) (20 Gy vs 20 Gy + IMT, *p = 0.5685*; 30 Gy vs 30 Gy + IMT, *p = 0.5500)*. Together, these findings do not support the hypothesis that the direct telomerase inhibition will sensitize cancer cells to IR hence making radiation more effective at lower doses, thus minimizing the devastating effects of the radiation therapy.

## Discussion

Medulloblastoma (MB) accounts for approximately 25% of all brain tumors in children. Group 3 MB is refractory to multimodal therapy and displays the worst prognosis. Radiotherapy is a standard treatment in older patients with group 3 MB but is rarely curative and is associated with acute and long-term toxicities. Craniospinal RT, at a young age leads to devastating neurocognitive decline. Achieving a cure for children with poor-prognosis MB while minimizing radiotherapy-associated sequelae remains a major goal of pediatric neuro-oncology. Given the role played by telomerase reactivation in oncogenesis, telomeres and telomerase are relevant therapeutic targets in children with high-risk brain tumors. With the aim to improve radiation efficacy and minimizing the associated toxicities, we sought to sensitize MB tumors to radiation by using a direct telomerase inhibitor. We found here that IMT, used as a radiosensitizer, had a limited effect on tumor growth, recurrence, and survival. Similar results were observed using a murine orthotopic model of human glioblastoma (27). IMT, is the only telomerase inhibitor tested in adults and children (9, 28–30). We have conducted the first phase I and II clinical trials with IMT in children (9, 28). Our phase II clinical trial of IMT proved intolerable and ineffective in children with recurrent or refractory CNS malignancies due, at least in part, to toxicities that prevented more frequent dosing of IMT to allow sustained inhibition of telomerase (9). Specifically, that there were two deaths due to intracranial hemorrhage associated with thrombocytopenia. Importantly, IMT treatment led to in-tumor inhibition of telomerase activity, indicating that IMT crosses the blood–brain barrier. Targeting telomerase directly, would result in a significant lag period from the initiation of treatment until telomeres shortened sufficiently to reduce tumor burden, while stopping therapy with IMT would lead to rapid telomere regrowth. Therefore, for these reasons and based on our present findings, we do not recommend direct telomerase inhibition as a radiosensitization approach to treat high-risk pediatric brain tumors. We have tested a new approach of telomere targeting strategy consisting of the incorporation of 6-thio-2’-deoxyguanosine (6-thio-dG), a telomerase substrate precursor nucleoside analogue, into telomeres by telomerase (31). Because this effect appears to be telomere length independent, the prediction using this novel approach is that treatment with 6-thio-dG will require a shorter time period to achieve a rapid effect on tumor growth and progression than direct telomerase inhibition-based therapy (32).We recently tested the *in vitro* and *in vivo* effect of 6-thio-dG in telomerase-positive stem-like cells derived from poor-prognosis pediatric brain tumors (18). Treatment with 6-thio-dG induced persistent telomere dysfunction and cell death within days in all telomerase-positive cell lines tested. Furthermore, 6-thio-dG crossed the blood–brain barrier and could specifically targeted tumor cells in an orthotopic mouse model of diffuse intrinsic pontine glioma, another deadly tumor in children. Our findings documented that 6-thio-dG is a promising novel approach to treat therapy-resistant pediatric brain tumors and provided a rationale for 6-thio-dG testing as a single agent or in combination with radiotherapy.

## Data availability statement

The raw data supporting the conclusions of this article will be made available by the authors, without undue reservation.

## Ethics statement

The animal study was reviewed and approved by IACUC2015-0066, Cincinnati Children’s Hospital Medical Center.

## Author contributions

Conception and design: RD. Development of methodology: SS, RD. Acquisition of data: SS, SK, MS, KB. Analysis and interpretation of data: SS, SK, KB, XM, RD. Writing, review, and/or revision of the manuscript: SS, SK, RD. Administrative, technical, or material support (i.e., reporting or organizing data, constructing databases): SS, SK, MS, XM, RD. Study supervision: RD. All authors contributed to the article and approved the submitted version.

## Supplementary material

Supplementary Figure 1 Effect of IR and IMT, and IR on in-tumor telomerase activity. Quantification of telomerase products were normalized with the internal control (IC) using Image Studio Lite (LI-COR Biosciences) and represented as bar graphs in arbitrary units (AU). Error bars represent the SD between different collected tumor samples (n=3-7 for each treatment arm). Multiple comparisons were conducted using One-way ANOVA and corrected using the Tukey method. * *p*< 0.0332; ** *p*< 0.0021; *** *p*< 0.0002, **** *p*< 0.0001.
