# Supplementary figures and images for: Limitations of radiosensitization by direct telomerase inhibition to treat high-risk medulloblastoma
Source: Front Oncol. 2023 Jan 18;13:1104670. doi: 10.3389/fonc.2023.1104670 (PMC9891285; doi:10.3389/fonc.2023.1104670)

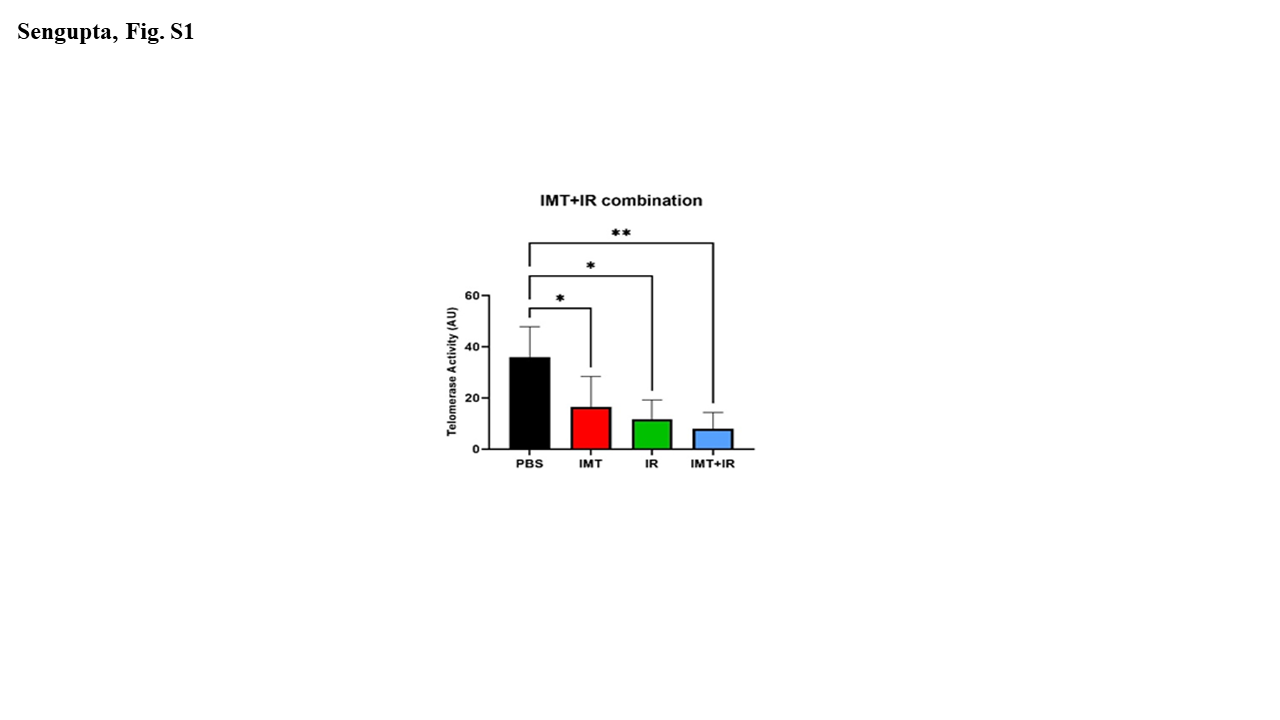

Supplement: Supplementary Figure 1 — Effect of IR and IMT, and IR on in-tumor telomerase activity. Quantification of telomerase products were normalized with the internal control (IC) using Image Studio Lite (LI-COR Biosciences) and represented as bar graphs in arbitrary units (AU). Error bars represent the SD between different collected tumor samples (n=3-7 for each treatment arm). Multiple comparisons were conducted using One-way ANOVA and corrected using the Tukey method. * p< 0.0332; ** p< 0.0021; *** p< 0.0002, **** p< 0.0001. [file Image_1.tif]
